# Assessing anthropogenic risk to sea otters (*Enhydra lutris nereis*) for reintroduction into San Francisco Bay

**DOI:** 10.7717/peerj.10241

**Published:** 2020-11-17

**Authors:** Jane Rudebusch, Brent B. Hughes, Katharyn E. Boyer, Ellen Hines

**Affiliations:** 1 Estuary & Ocean Science Center, San Francisco State University, Tiburon, CA, United States of America; 2 Department of Geography & Environment, San Francisco State University, San Francisco, CA, United States of America; 3 Department of Biology, Sonoma State University, Rohnert Park, CA, United States of America; 4 Department of Biology, San Francisco State University, San Francisco, CA, United States of America

**Keywords:** Spatial risk assessment, InVEST, Adaptive management, Sea otter, Species reintroduction, San Francisco Bay, Marine mammals, Anthropogenic risk

## Abstract

Southern sea otters have been actively managed for their conservation and recovery since listing on the federal Endangered Species Act in 1977. Still, they remain constrained to a geographically small area on the central coast of California relative to their former coast-wide range, with population numbers far below those of the estimated optimal sustainable population size. Species managers have discussed reintroducing southern sea otters into parts of their historic range to facilitate sustained population growth and geographic range expansion. San Francisco Bay (SFB), historically home to several thousand sea otters, is one location identified as a candidate release site for these reintroductions. The return of sea otters to SFB could bring benefits to local ecosystem restoration and tourism, in addition to spurring sea otter population growth to meet recovery goals. However, this is a highly urbanized estuary, so sea otters could also be exposed to serious anthropogenic threats that would challenge a successful reintroduction. In light of these potential detriments we performed a spatially-explicit risk assessment to analyze the suitability of SFB for southern sea otter reintroduction. We looked at threats to sea otters specific to SFB, including: the impacts of vessel traffic from commercial shipping, high-speed ferries, and recreational vessels; environmental contaminants of methylmercury and polychlorinated biphenyls; major oil spills; and commercial fishing. Factors that influenced the relative threat imposed by each stressor included the spatio-temporal extent and intensity of the stressor and its mitigation potential. Our analysis revealed the complex spatial and temporal variation in risk distribution across the SFB. The type and magnitude of anthropogenic risk was not uniformly distributed across the study area. For example, the central SFB housed the greatest cumulative risk, where a high degree of vessel traffic and other stressors occurred in conjunction. The individual stressors that contributed to this risk score varied across different parts of the study area as well. Whereas vessel traffic, particularly of fast ferries, was a high scoring risk factor in in the north and central bay, in the south bay it was environmental contaminants that caused greater risk potential. To help identify areas within the study area that managers might want to target for release efforts, the spatially-explicit risk map revealed pockets of SFB that could provide both suitable habitat and relatively low overall risk. However in some cases these were adjacent or in close proximity to identified high-risk portions of habitat in SFB. This predictive suitability and risk assessment can be used by managers to consider the spatial distribution of potential threats, and risk abatement that may be necessary for sea otters to re-occupy their historic home range in SFB.

## Introduction

When planning conservation actions, the complex ways in which humans influence wildlife and habitat suitability through direct and indirect actions must be considered (Ditchkoff, Saalfeld & Gibson, 2006). For marine mammals, the greatest threats to their survival stem directly and indirectly (e.g., loss of prey through overfishing) from anthropogenic sources (Jackson et al., 2001; Avila, Kaschner & Dormann, 2018). These threats include: incidental take in fisheries, vessel collisions, commercial hunting, pathogens, resource extraction, and alterations to the biophysical environment through habitat destruction, pollutants, or global climate change (Lotze et al., 2006; Davidson et al., 2012; Avila, Kaschner & Dormann, 2018). Coastal waterways are also often areas of concentrated urban development and population growth, due to the benefits of easy access to maritime travel, fishing, and commerce (Stewart et al., 2010). As a result, the coincidence of marine mammals and humans in these shared coastal habitats imposes distinct anthropogenic threats to wildlife and resources they utilize.

**Figure 1:**
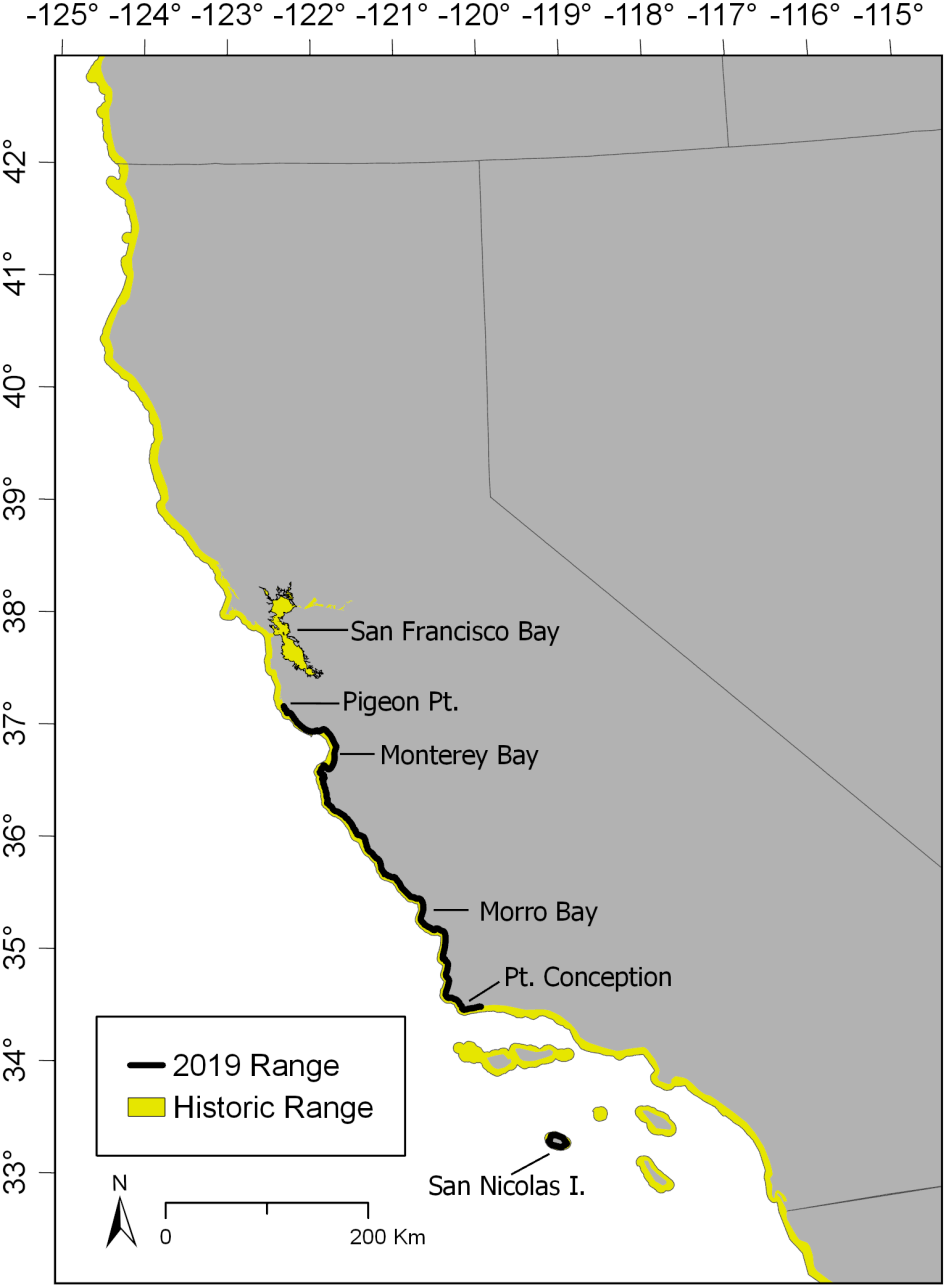
Historic and contemporary range extent of the southern sea otter. Yellow colored areas indicate the known distribution of southern sea otters in northern Baja Mexico and the western United States prior to their systematic removal during the fur trade between 1780–1840. The solid black line indicates the official range extent of southern sea otters as of 2019.

Prior to 1750, sea otters (*Enhydra lutris*, Linnaeus 1758) were found abundantly in the contiguous nearshore environments along the North Pacific Rim starting along the Pacific coast of Baja California, Mexico, up to Alaska, and around to Russia and Japan. Extensive hunting for their fur in the 18th and 19th centuries reduced the estimated 150,000–300,000 individuals into small, geographically isolated populations scattered in fragments of their historic range and totaling less than 2,000 individuals (Kenyon, 1969). Recovery of sea otters has advanced to varying degrees since protection under the International Fur Seal Treaty Act in 1911, but significant conservation challenges persist (Bodkin, 2015). Despite decades of protection under federal and California state legislation, the population of southern sea otters (*E. l. nereis*, Fig. 1) remains just over 3,000 individuals as of 2019 (Hatfield et al., 2019). By comparison, a conservative estimate of the maximum sustainable population of sea otters for the entire California coastline is 16,000 individuals (Laidre, Jameson & DeMaster, 2001). Southern sea otters currently occupy only 13% of their historic geographic range, and have not considerably expanded this range since 1998 (“Stock Assessment Report”, 2017; Tinker & Hatfield, 2017; Hatfield et al., 2019).

Curtailment of geographic range is considered one of the most significant challenges that hinder the recovery of sea otters in California. The cause of this lack of range expansion is likely due to a high degree of mortality from non-consumptive bites from white sharks (*Charcharodon carcharias*) at the northern and southern limits of the southern sea otter range (Tinker et al., 2015), causing regional population declines of 1.62% to 8.72% per year at the range peripheries in 2019 (Hatfield et al., 2019). As small marine mammals with limited diving capacity, southern sea otters are largely constrained to occupying waters where they can access benthic prey in depths no greater than 40 m (Bodkin, Esslinger & Monson, 2004; Thometz et al., 2016). Depth drops quickly with distance from shore along the central California coast; hence, southern sea otters move linearly along the shoreline, usually within 2 km from shore, avoiding the deeper offshore habitat (Tarjan & Tinker, 2016; Tinker et al., 2019a). This depth limitation requires that sea otters be able to expand their coastal range north and south into new, resource-abundant territories in order to achieve long-term sustainable population growth. Because southern sea otters have not been able to do this on their own, the US Fish and Wildlife Service and conservation organizations are looking to facilitate this range expansion through sea otter reintroduction efforts in other parts of their historic California range (A Johnson, 2018, pers. comm.; L Carswell, 2019, pers. comm.). Following the successful model of population enhancement by Mayer et al. (2019) of sea otters in Elkhorn Slough, several locations, primarily the coastal estuaries of California—including San Francisco Bay—have been identified as potential release sites (A Johnson, 2018, pers. comm.; L Carswell, 2019, pers. comm.).

Recently, more attention is being paid to the important role estuaries play in the ecology of southern sea otters and how these coastal embayments may play an important role in species recovery (Hughes et al., 2019). Compared to their well-studied use of coastal habitat, less is known about estuarine habitat use by southern sea otters as they currently occupy only two estuaries in the state. These two estuaries (Elkhorn Slough, Monterey County and Morro Bay, San Luis Obispo County) offer good evidence that estuaries provide important habitat for sea otters as they did in the past (Jones et al., 2011; Hughes et al., 2019). For example, sea otters recolonized Elkhorn Slough in 1984, and it now supports the highest concentration of sea otters in the southern sea otter range with roughly 100 resident sea otters (3% of the total population) in the small, 11 km long estuary (Kvitek et al., 1988; Maldini et al., 2012; Tinker & Hatfield, 2017). The contemporary success of sea otters in these two California estuaries and evidence of widespread utilization of estuaries in the archaeological record has motivated interest in targeting these spaces for sea otter recovery (Eby et al., 2017; Hughes et al., 2019). As the largest estuary on the Pacific coast of North America, the size of San Francisco Bay (400,000 hectares) has the potential to foster profound population growth for southern sea otters. Preliminary modeling of population growth and carrying capacity estimates that San Francisco Bay could support a population of sea otters 1.5 *times* the size of the current range-wide population (Hughes et al., 2019). While San Francisco Bay seems a natural choice for facilitating the future recovery of southern sea otters because of the quantity of sea otters it could support, there are challenges to living in this urban estuary that must not be overlooked.

Despite historic occupancy, sea otters have not continuously inhabited San Francisco Bay since the mid-19th century, after being extirpated during the Maritime Fur Trade (Ogden, 1941). Today San Francisco Bay is host to three major metropolitan cities, bayfront crude oil refineries, fast passenger ferries (median speed > 55 km/h), and five commercial shipping ports—some of the busiest in the world (Grobar, 2008; Jensen et al., 2015; Cope et al., 2020). There is an ever-present risk of large oil spills, historically proven to be devastating events for all kinds of wildlife (Garrott, Eberhardt & Burn, 1993; Peterson et al., 2003). In particular, oiling of sea otters damages their pelts and their ability to insulate, preventing them from thermoregulating and resulting quickly in hypothermia (Costa & Kooyman, 1982). An examination of deceased otters from the *Exxon Valdez* oil spill found that exposure to oil through ingestion or inhalation as a result of grooming to try and rid their fur of oil resulted in pathological lesions in the lungs, liver, and kidneys, and provoked a stress response that lead to shock and consequent mortality (Lipscomb et al., 1994). In addition, the Bay is popular for recreational boating and fishing, making the waterways of San Francisco Bay heavily trafficked by vessels of all types (Cope et al., 2020). There are few limits on the speed of vessels traveling within the Bay, apart from a 15 knots (27.8 km/h) speed limit imposed on large commercial shipping vessels while navigating the shipping channels (Harbor Safety Committee, 2009). Vessels pose a number of threats to sea otters as they do to other marine mammals, particularly when traveling at high speeds. For example, collisions between cetaceans and vessels resulted in greater likelihood of death as the outcome if the vessel was transiting at speeds greater than 10 kn (18.5 km/h) (Laist et al., 2001). Both legacy and emerging environmental contaminants are sequestered in the sediments of the Bay (Davis et al., 2007; Gehrke, Blum & Marvin-DiPasquale, 2011; Klosterhaus et al., 2012). Environmental contaminants (*e.g*., methylmercury, and polychlorinated biphenyls [PCBs]) have been documented to readily bioaccumulate in the tissues of sea otters and other marine mammals and have been tenuously linked to immunosuppression (Jessup et al., 2010; Desforges et al., 2016). Together these activities, in combination with the major loss of historic wetland habitat could make recovery of sea otters in San Francisco Bay a challenging endeavor.

Southern sea otters returning to San Francisco Bay will encounter an ecological landscape fundamentally altered by anthropogenic activities since they previously occupied the estuary over a century ago. When evaluating this site for reintroduction potential, understanding how these stressors affect the ability of sea otters to successfully inhabit San Francisco Bay is a critical component of the decision-making process. One technique that can be used to help bridge this knowledge gap is spatial risk assessment. Risk assessments allow for rapid, broad scale identification and evaluation of threats to the livelihoods of individuals, populations, or ecosystems (Gibbs & Browman, 2015). Risk assessments have been employed in the marine environment to analyze the hazards posed to a variety of marine mammals from both singular anthropogenic sources (e.g., bycatch, vessel traffic), and cumulative impacts from multiple sources (Davidson et al., 2012; Redfern et al., 2013; Brown, Reid & Rogan, 2015). Here, we take an integrative approach to predictive habitat suitability analysis by incorporating the contributions of anthropogenic stressors on habitat quality to answer several questions regarding the potential for San Francisco Bay to function as suitable habitat for southern sea otters:

1. What factors pose significant risk to sea otters in San Francisco Bay?
2. How are these stressors spatially and temporally distributed throughout San Francisco Bay?
3. How might these stressors threaten the quality and availability of sea otter habitat and impede recovery efforts in San Francisco Bay?

## Materials & Methods

### Study area

**Figure 2:**
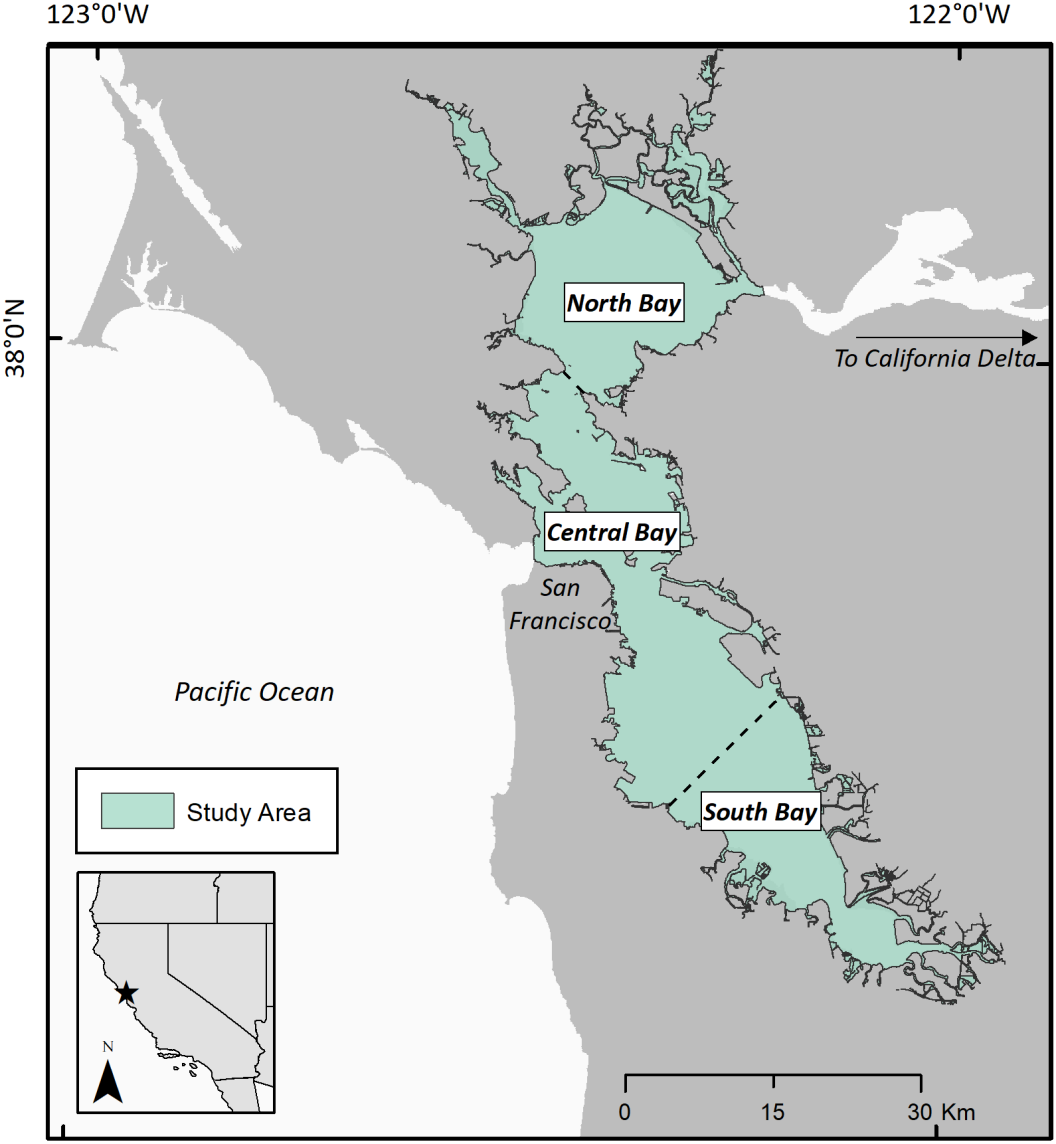
San Francisco Bay study area. Our study area of San Francisco Bay and the three sub-regions of the study area: north, central, and south bay.

San Francisco Bay is a shallow estuary situated on the north-central coastline of California. The Bay receives marine water tidally from the Pacific Ocean and seasonally fluctuating freshwater flow from the inland watersheds that drain through the Sacramento-San Joaquin Delta into the Bay (Nichols et al., 1986) (Fig. 2). Intense urbanization since the local extirpation of sea otters has caused dramatic changes to habitat and water quality in San Francisco Bay leading to less acreage of potentially suitable habitat. Since 1849, over 95% of the tidal marshes within San Francisco Bay have been degraded or destroyed as a result of agriculture and urban and industrial uses (Brophy et al., 2019). Eelgrass in the Bay covers an order of magnitude less acreage than predicted as suitable habitat (Merkel & Associates, 2005). Restoration efforts are struggling to reestablish eelgrass beds, in part due to persistent anthropogenic threats (Boyer & Wyllie-Echeverria, 2010). Despite the heavy influence of anthropogenic alterations and land use conversion, the Bay supports an array of habitat types, including tidal marshes and submerged aquatic vegetation (Atwater et al., 1979). Many animal species rely on the Bay ecosystems during part or all of their lifecycles, including invertebrates, anadromous fish, birds, and terrestrial and other marine mammals, such as Pacific harbor seals and California sea lions, harbor porpoise, and larger cetaceans such as humpback and gray whales (Josselyn, 1983; Keener et al., 2016; Stern et al., 2017).

### Habitat data

First, we divided the Bay into the north, central, and south bay sub-regions to comparatively assess spatial variation in the distribution and intensity of anthropogenic risk factors. We then determined the extent of potential sea otter habitat present for each sub-region by mapping five habitat types: open water (nearshore), open water (offshore), eelgrass (*Zostera marina*), intertidal mudflats, and salt marsh. Areas in our study region that met the primary criteria for defining sea otter habitat (<40 m deep) were classified as “open water: nearshore” or “open water: offshore” depending on whether they were within or further than 2 km from shore, respectively. It is unknown to what extent sea otters would utilize waters that are greater than 2 km from shore, yet shallow enough to dive easily. Modeling analysis of southern sea otter distribution in soft-sediment nearshore vs. offshore waters in other parts of California found that sea otter occupancy of waters greater than 2 km from shore was just 15-percent of their utilization of waters closer than 2 km from shore (Hughes et al., 2019). Studies from northern sea otter (*E. l. kenyoni*) populations in Southeast Alaska indicate that shallow offshore habitats likely would be utilized at least to some extent by southern sea otters, and so we included these areas in our assessment of potential habitat in San Francisco Bay (Esslinger & Bodkin, 2009; Tinker et al., 2019b). We also included salt marshes (*i.e.,* vegetated marsh banks, and associated tidal creeks within the marsh), intertidal mud flats, and beds of eelgrass (California Aquatic Resources Inventory, 2017).

### Stressor spatial data

We chose seven anthropogenic stressors that pose a potential threat to sea otters: (1) commercial shipping, (2) ferries, (3) recreational vessels, (4) large oil spills, (5) methylmercury, (6) Polychlorinated Biphenyls (PCBs), and (7) commercial fishing. The selection of these stressor types was made in consultation with sea otter species managers, who directed us to these categories as being of particular concern. Using a geographic information system (GIS), we utilized existing data sets (Table 1) to create spatial footprints of each stressor’s presence in the study area. Vessel traffic data collected by the on-board Automatic Identification System (AIS) devices from 2014 (the most current available) were downloaded and the relevant vessel classes were extracted by their AIS vessel type: cargo ships and tankers (which we grouped as “shipping vessels”), passenger (...“ferries”), and pleasurecraft/sailing (...“recreational vessels”) (See Supplemental Information for details on these AIS vessel types and codes). This resulted in a dataset of over 2 million data points representing the exact locations of these vessels. These were then transformed into density rasters and then converted to presence/absence shapefiles inputted into the HRA. Oil spilled into the marine environment could feasibly travel anywhere, so we relied on the spatial footprints of cargo ships and crude oil tankers as the most likely source of (and the area most immediately impacted by) a large oil spill. Methylmercury data was represented by concentration in water (the bioavailable form), and PCBs were measured as concentrations sequestered in sediment from 2006-2014. The processes of biomagnification and bioaccumulation are complex. Without empirical evidence relating concentrations of environmental contaminants in situ to threshold levels of contamination in sea otters, our contaminant data does not necessarily represent biologically significant amounts for sea otters. We instead selected the upper 60th percentile of values for each to capture notably elevated levels of contaminants and created spatial footprints from those values (0.09–0.15 ng/L for methylmercury and 10.9–18 ppb for PCBs). Data for commercial fishing effort spanned across 5 years (2012–2017) in order to capture interannual variation in herring spawn and fishing effort locations.

**Table 1:** Anthropogenic stressors in San Francisco Bay chosen for the study, potential risks posed by each of the stressors to sea otters, and sources of the data used.

Stressor	Description	Risk(s) attributed	Data type & source
Vessel Traffic	Passenger ferries, cargo ships, tankers, small recreational craft (i.e., sailboats, motorized boats)	Behavioral disturbance, injury or death caused by vessel collisions	AIS vessel positions, collected during 2014 Bureau of Ocean Energy Management, National Oceanic and Atmospheric Administration, U.S. Coast Guard Available from: https://marinecadastre.gov/ais/
Contaminants	PCBs, methylmercury	Immunosuppression	Contaminant samples in sediment from 2002–2014 San Francisco Estuary Institute Available from: https://cd3.sfei.org/
Commercial Fishing	Activities and gear, including set gillnets, associated with seasonal herring fishery	Behavioral disturbance, bycatch	Herring spawning location data from 2012-2017 California Department of Fish and Wildlife Available from: https://data.cnra.ca.gov/dataset
Oil Spills (Major)	Petroleum products unintentionally entering the marine environment from man-made sources	Oiling of fur leading to hypothermia; liver, kidney, and lung lesions, gastric hemorrhaging, shock, death	AIS cargo ship and crude oil tanker positions, collected during 2014 Bureau of Ocean Energy Management, National Oceanic and Atmospheric Administration, U.S. Coast Guard Available from: https://marinecadastre.gov/ais/

### Risk analysis

We used the open-source tool, Habitat Risk Assessment (HRA) (InVEST v.3.5.0), to calculate the cumulative risk potentially incurred by sea otters living in San Francisco Bay as a consequence of anthropogenic stressors. We defined a stressor as any extrinsic activity or source having a negative impact on sea otter health, via behavioral modification, illness, injury, or death (EPA, 2020). Because sea otters do not currently occupy San Francisco Bay, we used habitat suitability as a proxy for species presence. The HRA model uses an exposure—consequence methodology to evaluate threats posed by anthropogenic activities to marine environments (Arkema et al., 2014). Exposure is the degree of spatial and temporal overlap of a given stressor onto a habitat, as measured by four criteria: spatial overlap, temporal overlap, intensity, and management effectiveness. The response of a habitat or species to that exposure is the consequence of exposure to a stressor, represented by two criteria: change in area and intensity. The HRA calculates spatial overlap of each stressor onto the study area and, where overlap occurs, calculates exposure and consequence values using the risk criteria. If a stressor does not overlap with the habitat, it is assumed that there is no risk because there is no exposure of the habitat to the stressor and therefore no consequence of exposure. After calculating exposure and consequence values, the model performs a Euclidean Risk calculation by combing the exposure and consequence values for each habitat and stressor combination, generating a cumulative risk score across the study region within a grid of cells (resolution = 100 m). Risk to each study sub-region is also generated in this way, but within the bounds of the defined sub-regions. The sub-regional scores are the averaged exposure and consequence scores within the sub-region and risk is calculated using the same Euclidean Risk method. Full documentation and equations for the HRA risk calculations can be found in the InVEST HRA User Guide (InVEST, 2017).

### Stressor weighting

Numerical weights were applied to each stressor in the model to adjust the relative importance of that stressor, making the HRA flexible to the inputs and modifications done by the user. To objectively assign these weights, we solicited expert opinion through one-on-one key informant interviews with individuals (*n* = 10) identified as holding specific expert knowledge of sea otter biology. The survey consisted of four questions pertaining to the response of sea otters to each stressor as related to the risk criteria of the HRA (Table 2). Participants were asked to categorize the potential threats posed by each stressor, and the role location and temporality played in influencing the level of threat. The objective was to characterize the nature of the impact each stressor could have on sea otter livelihood by assigning a numerical value (1 to 3) to each stressor, corresponding to the low-to-high weighting scheme used by the HRA. Participants were also encouraged to elaborate on the reasoning behind their responses to further supplement our understanding and interpretation of the relative significance of each stressor. From these interviews, we generated a final weighting scheme used to calculate risk in the model by averaging the scores given to each stressor across all participants. If scores didn’t average out to a whole number, they were rounded to the nearest whole number. When respondents expressed low confidence in their response to a question, we still included their data and encouraged them to come up with a concrete numerical value for each weight. In some cases, respondents answered with a range of values (i.e., “1 or 2”). For these, we calculated averages twice using the low-end and high-end values, but the two different averages resulted in the same weight after rounding to the nearest whole number regardless. To account for levels of uncertainty related to data quality, the HRA includes a category for weighting data quality based on Best Data (1), Adequate Data (2), or Limited Data (3) (InVEST, 2017). We scored all criteria a data quality score of 2, defined by the HRA as information based on knowledge or data collected outside the study region or on a closely related species and may have limited supporting empirical evidence.

**Table 2:** Risk Criteria. Habitat Risk Assessment (HRA) risk criteria, the definitions of each criteria category and the definitions of each weighting category.

Criteria	Definition	Weights		
		Low (1)	Medium (2)	High (3)
Temporal Overlap	How often is the species exposed to a stressor?	Annually to less than annually	Weekly to monthly	Daily to several times per day
Change in Area	What portion of the habitat area is altered or rendered unavailable as a result of a stressor?	0–33%	33–66%	66–100%
Intensity	How severe is the response of species to a stressor?	Results in minor behavior change	Results in major behavior change or injury	Results in death of one to several sea otters
Management Effectiveness	How effective are management actions at reducing threat of a stressor?	Very effective	Somewhat effective	Not effective/not able to be managed

### Risk criteria

#### Spatial overlap

Spatial overlap was defined as the extent to which a stressor occupies the same geographic space as a habitat (Arkema et al., 2014). Spatial overlap was the first criteria evaluated by the HRA. First, a binary method was used to assign cells containing either no overlap of a habitat and stressor (spatial overlap = 0) or overlap of a habitat and stressor (spatial overlap = 1). In cells where spatial overlap occurred, risk exposure was then calculated using the other four risk criteria (InVEST, 2017).

#### Temporal overlap

Temporal overlap is an exposure criterion that refers to the frequency with which a habitat is exposed to a stressor (Arkema et al., 2014). Temporality of each stressor was already assigned with the stressor datasets used, with activities ranging in occurrence from multiple times per day to less than once per year (Table 2). In assigning weights for temporal overlap, we assumed that the more frequently a stressor occurred in the study area, the more exposure sea otters would have. Weights were assigned to each stressor on a scale from 1 to 3, where 1 denotes low/episodic/infrequent stressor occurrence in the study area (no more than 1 occurrence per year), 2 indicates moderate/frequent stressor occurrence (monthly), and 3 denotes high frequency stressor occurrence, daily to several times per day.

#### Change in area

The degree to which habitat availability is decreased as a result of a stressor is reflected in the HRA by the change in area consequence criterion (Arkema et al., 2014) (Table 2). We calculated the percent area reduced by each stressor by dividing the maximum spatial extent of each stressor by the area of the study region. We then converted this percentage into a change in area criteria weight on a 1–3 scale. Stressors that overlapped between 0–33% of the study area received a rating of 1, those that had overlap between 33–66% of the study area received a rating of 2, and those that overlapped 66–100% of the study area received a rating of 3.

#### Intensity

The intensity criterion rates the severity of impact caused by a stressor to sea otters (Table 2). We relied on expert opinion to supplement data gaps on the effects each stressor might have on sea otters. We considered the level of threat posed by each stressor to range on a spectrum from sub-lethal (behavioral disturbance or injury) to lethal and asked experts to identify the most likely response each stressor would illicit.

#### Management effectiveness

Management effectiveness is an exposure criterion that describes the degree to which negative effects of a stressor can be controlled or mitigated by management action (Arkema et al., 2014). We asked each expert if there were protocols to manage the harmful effects of each stressor based on practices currently or historically used with sea otters (Table 2). Stressors that could be effectively managed received a lower weighting in the model than those that could not be managed or managed with difficulty. Context is an important consideration in ascribing management potential in stressors. If a stressor had been managed to reduce impact to sea otters elsewhere, but the feasibility of implementing the same procedure in San Francisco Bay was low, we ascribed lower management potential (higher criteria rating) to that stressor.

## Results

### Habitat

**Figure 3:**
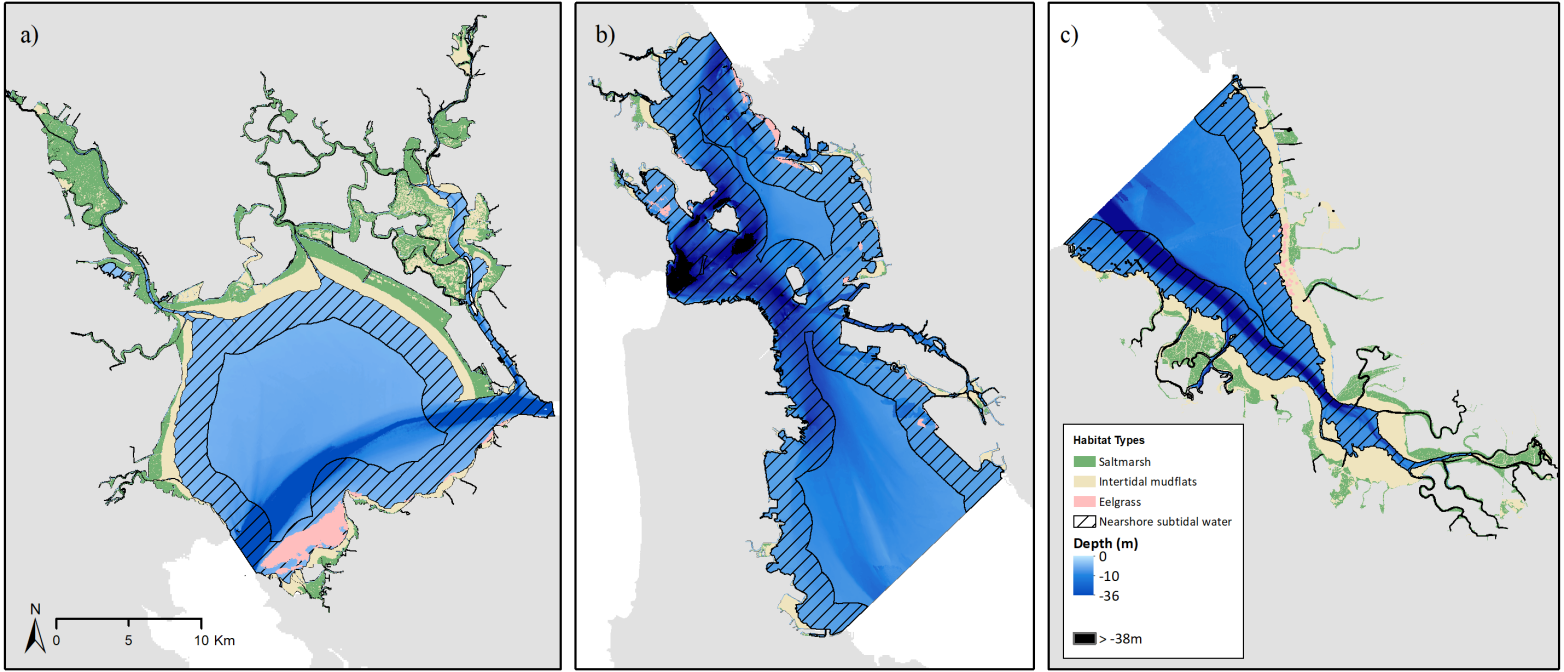
Potential sea otter habitat in the San Francisco Bay. Presence of potential sea otter habitats determined within the study area across the (A) north, (B) central, and (C) south bay subregions. Salt marsh habitats are shown in green, intertidal mud flats shown in beige, eelgrass beds shown in pink, and subtidal water shown with bathymetry in blue. Hatched areas indicate nearshore (<2 km from shore) waters. Black areas indicated water where depth is ≥39 m.

Estuarine habitats likely suitable for sea otters were present throughout the study area, but varied in spatial extent throughout each sub-region (Figs. 3A–3C; Table 3). The largest areas of salt marsh occur in the North and South Bay regions, while the Central Bay has the least amount present. Collectively, saltmarsh covers 706.7 km^2^ (60.3%) of the total study area. All areas of subtidal water in the North and South Bays, and nearly all in the Central Bay, were less than 40 m deep. Nearshore water covered 552.9 km^2^ in total area, and offshore area totaled 33 km^2^. The extent of eelgrass throughout San Francisco Bay was proportionately very low, covering less than 1% of the total study area (or 8.5 km^2^).

**Table 3:** Habitat. The extent of estuarine habitat types present in San Francisco Bay, as a proportion of the total area of each subregion.

Study Region	North	Central	South
*Total Area* (km^2^):	387	469	289
*Habitat Type and proportion (%) of total subregion area:*			
Subtidal Water: Nearshore	36.7	64.0	38.4
Subtidal Water: Offshore	31.0	31.4	24.9
Saltmarsh	18.6	<1.0	13.6
Eelgrass	<1.0	<1.0	<1.0
Intertidal Mud	13.2	3.1	22.7

### Risk criteria & stressor weighting

#### Spatial overlap

The HRA analysis revealed differences in the spatial extent of stressors across the regions of the San Francisco Bay study area. In the North Bay, vessel traffic posed the greatest risk, whereas environmental contaminants (PCBs and methylmercury) posed the greatest risk to sea otters in the South Bay. The Central Bay region had a mix of both vessel traffic and environmental contaminants that posed risk to sea otters. Values calculated by the HRA for spatial overlap can be found in Table 4.

**Table 4:** Anthropogenic Stressor Ratings. Final criteria ratings for each stressor used by the Habitat Risk Assessment tool to determine total risk. Values range from no risk (0), low (1), medium (2) to high (3) risk. Spatial overlap criteria were given for each individual sub-region: north bay (NB), central bay (CB), south bay (SB).

Stressor	**Exposure criteria**			**Consequence criteria**	
	Spatial overlap NB/CB/SB	Temporal overlap	Management effectiveness	Change in area	Intensity
Vessel Traffic-Ferries	1.1/1.5/0.0	3	3	1	3
Vessel Traffic- Shipping	1.1/1.4/1.1	3	3	1	1
Vessel Traffic-Recreational	1.8/2.6/1.7	3	3	2	1
Contaminants-Methylmercury	1.1/1.9/2.4	3	2	2	2
Contaminants-PCBs	0.0/2.4/2.8	3	2	2	2
Commercial Fishing	1.0/1.2/1.0	2	3	1	1
Oil Spills	1.2/1.7/1.2	1	1	1	3

#### Temporal overlap

The most frequent stressors sea otters could be exposed to were vessel traffic and contaminants (Table 4). All vessel traffic and contaminant stressors received a ranking of 3 as they were present a near-constant presence in the study area. Commercial fishing, present only during the open season between January 1 and March 15, was assigned a weight of 2 accordingly. Large oil spills were a rare occurrence, happening less than annually, and were assigned a weight of 1.

### Change in area

The two stressors that posed the greatest risk to available sea otter habitat were recreational vessels (57.8% of available habitat) and PCBs (52.9% of available habitat). Using the spatial footprint of each stressor we calculated the percent area reduced by each stressor in the study area (Table 4). Our calculations showed that ferries occupied 12.9% of the study area, cargo and tanker ships occupied 12.2% and recreational vessels occupied 57.8%. Areas where high concentrations of methylmercury occurred in sediment were across 50.1% of the study area and PCBs across 52.9% of the area. Commercial fishing occurred in 3.4% of the study area. The threat of a large oil spill covered 30.1% of the study area.

### Intensity

Experts were in agreement that among the stressors included in our analysis, vessel traffic, and in particular speed of vessel, was of primary concern (Table 4). We assigned commuter ferries, the faster vessels, an intensity ranking of 3 as these vessels travel upwards of 38 kn (78 km/h) in the study area (Cope et al., 2020). The primary threat attributed by experts to slower-moving vessels, including recreational craft, cargo, and tanker ships, was change of behavior and each was scored an intensity rating of 1. Experts were highly conservative in rating the criterion for contaminants due to gaps in knowledge about the effects on sea otters. They concluded that the primary consequence of this stressor was likely to be declined health, but not necessarily direct mortality, and conservatively assigned a ranking of 2 to both methylmercury and PCBs. Experts cited bycatch in the commercial herring fishery as a potential threat, as sea otter bycatch in southern California gillnet fisheries was an issue historically (Wendell, Hardy & Ames, 1986). However, experts also felt that, given the mesh size and method of setting the gear in this particular herring fishery, sea otters were not likely to get entangled in the nets. The more likely impact of fisheries activities was considered to be behavioral disturbance, so we assigned fishing an intensity ranking of 1. Experts agreed that the primary threat from a large oil spill was mortality and received an intensity ranking of 3. Oil and other petroleum products can spill in different quantities with varying degrees of impact to wildlife, so we asked experts to contextualize this threat in terms of a large oil spill, using the most recent such spill in San Francisco Bay (the 53,569-gallon *M/V Cosco Busan* spill in 2007) as an example.

### Management effectiveness

According to experts, restrictions on vessel speed to below 5 knots (9.2 km/h) have been shown to lessen the likelihood of vessel collisions with sea otters. However, because minimal vessel speed restrictions are currently established in San Francisco Bay, they considered this stressor category to not be managed within the study region (Table 4). We referred to the San Francisco Bay Regional Water Quality Control Board (https://www.waterboards.ca.gov) to determine whether methylmercury or PCB contamination was currently being managed in San Francisco Bay. Clean Water Action Plans (TMDLs) for both mercury and PCB’s exist for the San Francisco Bay region, mandating that industries take action to reduce these contaminants in the Bay. We scored both contaminants for management effectiveness as being ‘somewhat effective’ based on some implementation of programs to prevent further contamination by regulating sources in stormwater and wastewater discharge, but despite this there is continued persistence of legacy and emerging contaminants in the environment (Sutton et al., 2017). For oil spills, experts directed us to the Office of Spill Prevention and Response (OSPR), the Oiled Wildlife Care Network (OWCN), and the state oil spill contingency plan as examples of strategies in place that aim to prevent and respond to oil spills in California and recover and rehabilitate affected wildlife. In addition, lessons learned after past oil spills (e.g., *Exxon Valdez* in 1989), as well as research on oiled otter washing and rehabilitation (ex: Jessup et al., 2012), have greatly improved likely outcomes for oiled sea otters in California. The combination of prevention and response protocols in place led experts to conclude that agencies would be well prepared to respond and mitigate an oil spill in San Francisco Bay. They therefore considered an oil spill to be an effectively managed stressor.

### Risk analysis

**Figure 4:**
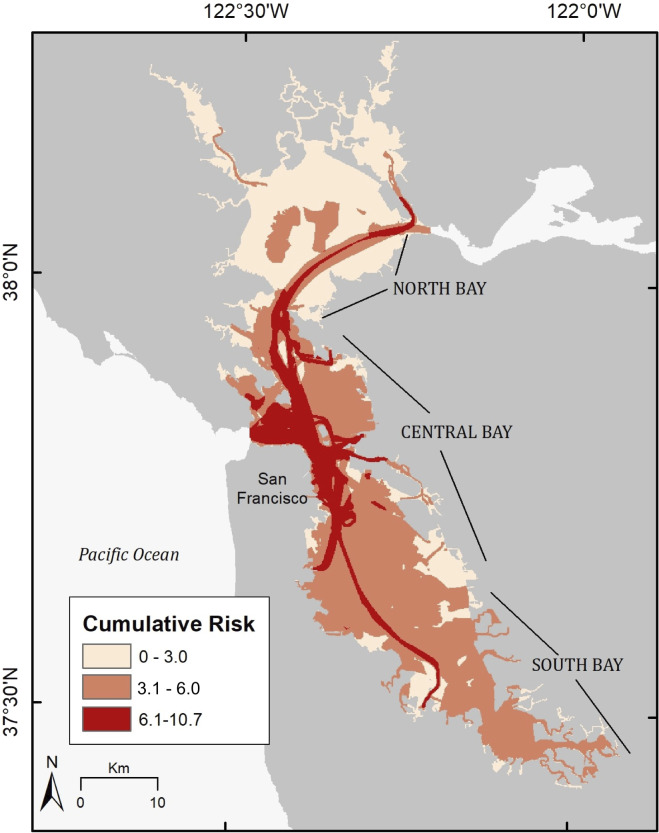
Cumulative risk map of anthropogenic risk distribution across the study area. Results of the Habitat Risk Assessment showing the spatial distribution of anthropogenic risk across the study area. Cumulative risk scores ranged from 0 to 10.7, out of a possible maximum cumulative risk score of 21. A gradient of color shades from light-to-dark red indicate the cumulative risk from low-to-high, respectively.

Risk from each anthropogenic stressor varied spatially across each sub-region of the study area. Cumulative risk from all 7 stressors was highest in the Central Bay and lowest in the North Bay. Some of the highest risk areas occurred adjacent to highly utilized urban hubs, for example the San Francisco waterfront and the waters around the Golden Gate Bridge (Fig. 4). Cumulative risk scores ranged from 0 to 10.7, with higher risk areas occurring where multiple stressors overlapped with one another. Given a total possible risk score of 21 (a case in which all stressors overlapped onto the study area and the maximum Risk score (*R* = 3) was achieved by all stressors), our maximum cumulative risk scores calculated approached roughly half of the maximum potential risk for the study area.

**Figure 5:**
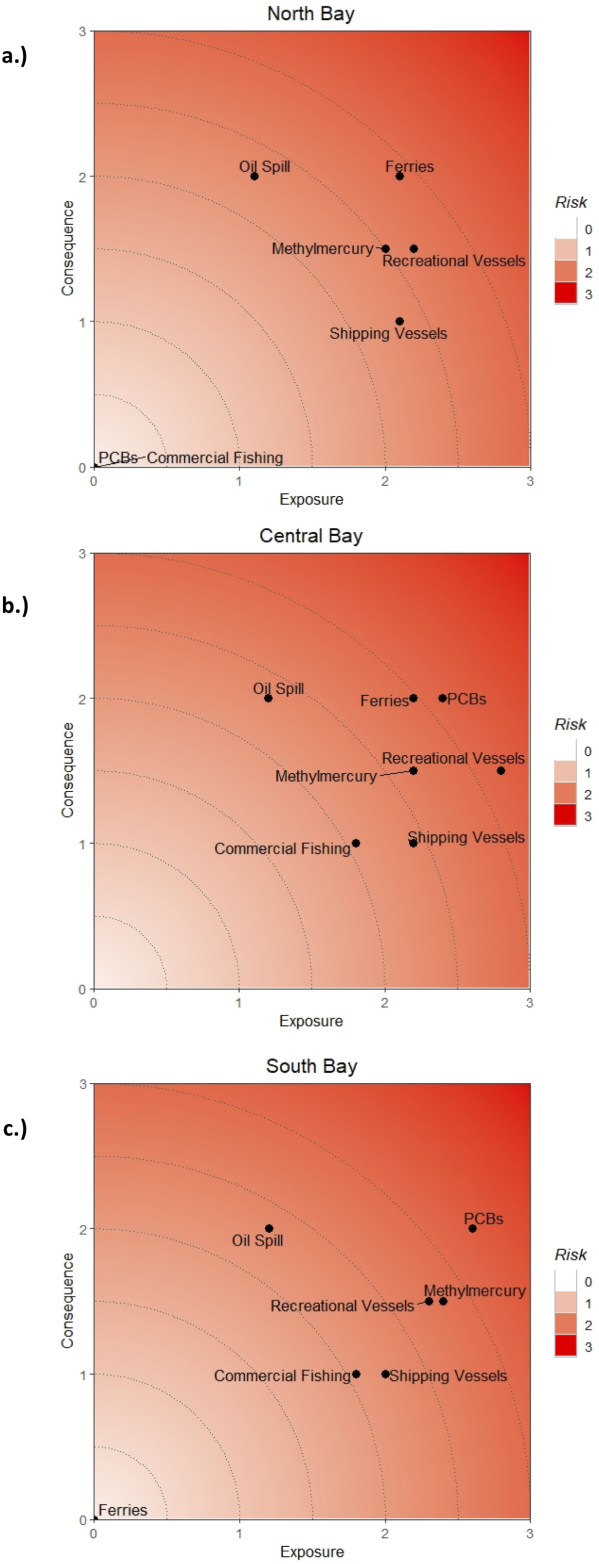
Risk plots of *Exposure* and *Consequence of Exposure* scores for the study area. Risk plots generated by the Habitat Risk Assessment showing each stressor plotted as a function of their *Exposure* and *Consequence of Exposure* values in the (A) North, (B) Central, and (C) South Bay subregions.

At the sub-regional level, the HRA model generated exposure, consequence of exposure, and risk scores (Table 5) for each stressor and risk plots (Figs. 5A–5C) for each sub-region. In the North Bay, the highest exposure came from vessel traffic of all types, followed by methylmercury contamination and commercial fishing. The two stressors scoring highest for consequence of exposure for this area were oil spills and ferries, followed by recreational vessels and methylmercury. Out of the seven stressors analyzed, two stressors (PCBs, commercial fishing) had no spatial overlap within the North Bay sub-region and thus had exposure and consequence scores of 0 (Table 5; Fig. 5A). In the Central Bay, all stressors had at least some degree of spatial overlap onto the sub-region. Recreational vessel traffic and PCBs had the highest exposure, followed by ferries, cargo and tanker ships, and methylmercury. The highest consequence of exposure came from oil spills, ferries, and PCBs (Table 5; Fig. 5B). In the South Bay, the highest exposure stressors were PCBs and methylmercury contaminants, and highest consequence was from PCBs and oil spills. Ferries had no occurrence in the South Bay sub-region and therefore had no risk score (Table 5; Fig. 5C).

**Table 5:** Habitat Risk Assessment calculated risk scores. Risk Scores from the Habitat Risk Assessment tool showing *Exposure* (E), *Consequence of Exposure* (C) and cumulative *Risk* (R) for each stressor across the three subregions of the study area.Risk Scores from the Habitat Risk Assessment tool showing Exposure (E), Consequence of Exposure (C) and cumulative Risk (R) for each stressor across the three subregions of the study area.

*Stressor*	North Bay			Central Bay			South Bay		
	***E***	***C***	***R***	***E***	***C***	***R***	***E***	***C***	***R***
Vessel Traffic-Ferries	2.1	2.0	1.5	2.2	2.0	1.6	0.0	0.0	0.0
Vessel Traffic- Shipping	2.1	1.0	1.1	2.2	1.0	1.2	2.0	1.0	1.0
Vessel Traffic-Pleasurecraft	2.2	1.5	1.5	2.8	1.5	1.6	2.3	1.5	1.4
Contaminants-methylmercury	2.0	1.5	1.1	2.2	1.5	1.3	2.4	1.5	1.5
Contaminants-PCBs	0.0	0.0	0.0	2.4	2.0	1.7	2.6	2.0	1.9
Commercial Fishing	0.0	0.0	0.0	1.8	1.0	0.8	1.8	1.0	0.8
Oil Spill	1.1	2.0	1.0	1.2	2.0	1.5	1.2	2.0	1.0

## Discussion

Our risk assessment fulfills a crucial step in an adaptive management framework to help inform decision-makers on the feasibility of sea otter reintroduction to San Francisco Bay by identifying potential hazards or conflicts posed by anthropogenic stressors. The areas of highest overall risk exposure were in the Central Bay sub-region, particularly around highly developed waterfronts and heavily trafficked waterways, e.g., the entrance of the Golden Gate and the Port of Oakland. The lowest risk exposure occurred in the North Bay sub-region where there was less co-occurrence of anthropogenic stressors. Additionally, our habitat analysis revealed that despite heavy degradation of the ecosystem, prime sea otter habitats remain present throughout the study area and are particularly abundant in the North and South Bays. We recommend focusing reintroduction efforts in areas of the Bay that offer the lowest exposure to risk and have supporting habitats. Coinciding release location with existing protected lands may further benefit the reintroduction process. Designated protected areas or ecological reserves may offer sea otters a refuge from chronic disturbance if they include areas off limits to people (Eby et al., 2017). In the North Bay, such areas include the San Pablo Bay National Wildlife Refuge and China Camp State Park, where risk from all anthropogenic stressors was low. In the lowest portion of the South Bay within the Don Edwards National Wildlife Refuge, low human activity and protected marsh habitat may provide suitable release locations as well. When possible, managers should also take advantage of collaboration with federal and state agencies that manage protected lands around the Bay as these partnerships could help to facilitate the reintroduction process and provide support for post-release monitoring efforts. In the two sub-regions (North and Central) where ferries were present, both exposure and consequence scores were high. Exposure scores for commercial shipping and recreational vessels were similarly high, although the consequence scores were lower. The spatial footprints of ferries and commercial shipping vessels revealed that these vessel types were confined to travel in very distinct paths through the study area on their way to various destinations.

Resiliency of a species to stressor exposure was not accounted for in our analysis, although experts indicated habituation as a possible response of sea otters to some of the stressors we analyzed. Habituation of an animal to a stressor can decrease the intensity of response elicited by that stressor, but it does not necessarily decrease risk exposure. In fact, animals that become comfortable with the presence of anthropogenic stressor in their environments can be exposed to risk in different ways, such as increased disease exposure or loss of an appropriate fear response in dangerous situations (Woodford, Butynski & Karesh, 2002). Bejder et al. (2009) emphasize that when habituation of wildlife to anthropogenic activities is incorrectly treated as a benign, or even positive behavioral response, it can lead to inappropriate conclusions about the impact these activities cause and thus the management of those activities. We urge that, when evaluating San Francisco Bay for reintroduction, species managers not consider habituation of sea otters to anthropogenic activities to be a desirable outcome, nor one that will necessarily reduce risk exposure.

Management actions that reduce the spatial extent of recreational vessel traffic would help lower the exposure score, and an education program on responsible recreational boating near sea otters could reduce the likelihood of behavioral disturbance. Exposure scores for contaminants were also high relative to consequence scores, although reducing the spatial extent of persistent legacy pollutants would be challenging. Consequence scores for oil spills were higher than exposure scores, indicating that while the effects of oiling were considered severe, the likelihood of exposure to oil is low in the study area due to the rarity of occurrence and effective mitigation protocols. Commercial fishing ranked consistently low for both exposure and consequence, suggesting that this activity presents less of a threat relative to the other stressors analyzed. Application of the Habitat Risk Assessment (HRA) to evaluate the reintroduction potential of a highly urbanize site presented both benefits and challenges. The HRA had the advantages of simplicity and efficacy, and the ability to incorporate a range of data types and quality while providing meaningful outcomes for conservation goals. The HRA also allowed the user to tailor the analysis to a local scale and determine the contribution of individual stressors to a specific risk landscape. This would in turn allow decision-makers to create targeted strategies of risk reduction or mitigation. The HRA relied heavily on expert opinion to calculate risk and therefore requires detailed knowledge of both the species being studied and how anthropogenic stressors might affect them. Collecting expert knowledge data through key informant interviews was especially useful as a low-cost and efficient method to make use of the best available science to fill gaps in the primary literature. Where empirical data were lacking, the interviews allowed us to extract quantitative values for the HRA risk criteria from the qualitative and finesse judgment of expert knowledge-holders. However, when gaps in expert knowledge were exposed during the interview process, in particular with regards to the effects of environmental contaminants on sea otters, our ability to accurately score the risk criteria for that stressor was limited. There was therefore a larger degree of uncertainty when interpreting the level of risk associated with those stressors.

As both habitat data and stressor spatial data in our study are refined and improved, the HRA’s risk predictions and usefulness will subsequently improve. We used the best available data for our study, however limitations to those datasets limited the scope of our ability to fully capture the spatial extent of some stressors. For example, our spatial datasets of vessel traffic were derived using vessel positions relayed through the Automatic Identification System (AIS), which is a device required to be used by some, but not all, classes of vessels. This means we were unable to include kayaks and other types of small personal watercraft in our analysis, which in other parts of the sea otter range cause significant behavioral disturbance to sea otters and exacerbate the already-high metabolic demand sea otters have (Barrett, 2019). In addition to these stressors that we were not able to include, many other potential stressors exist that should be considered further as well. These include biotoxins, pathogens from sewage runoff, recreational fishing, dredging, windsurfing and kite boarding, floatplanes, helicopters, or military drills, among others. For large oil spill risk, we relied only on the spatial footprints of the major sources of oil in the San Francisco Bay (crude oil tankers and cargo ships) but were not able to incorporate the movement of oil spilled into the environment in our analysis. Given the potentially devastating impact a large oil spill could have on sea otters and the local ecosystem, it would be a worthwhile avenue of further research to model the movement of oil within San Francisco Bay to more precisely identify areas prone to most risk. A large degree of uncertainty remains as to what level of impact environmental contaminants have on sea otter health. Both legacy and emerging environmental contaminants remain an issue of concern in San Francisco Bay, and while the data on a variety of contaminant levels and sources are available, it was apparent in both the literature and our expert opinion surveys that the actual impact contaminants have on sea otters is not fully understood. These relationships should be studied further in order to be able to fully assess the role of contaminants as actual stressors to sea otters and thereby allow managers to appropriately assess the quality of habitat San Francisco Bay provides.

It is not assumed that reintroduced sea otters will stay local to the areas they are released to. For this reason we kept our analysis at a large regional scale rather than focusing on one section of San Francisco Bay that may be perceived as ideal due to the existence of habitat or protection via a state or federal refuge. One of the benefits of our HRA analysis is the identification of areas where low anthropogenic risk would coincide with habitats thought to be more suitable to sea otters. These areas could then be targeted as places where the initial release of animals occurs, but managers should be careful to consider the threats posed by the greater surrounding areas because sea otters can and may potentially range widely from their release origin. While not a migratory species, sea otters are not sedentary animals either and are capable of transiting significant distances at times. In 2018, a sea otter that was released in Half Moon Bay, California following rehabilitation was re-found less than 12 h later and over 42 km north in San Francisco Bay, and then moved further north to the waters around Point Reyes National Seashore (S Johnson, 2019, pers. comm.). Keeping our analysis of San Francisco Bay’s risk landscape at a large regional scale ensured that we were able to encapsulate the full magnitude of potential risk and stressors that sea otters could encounter as they move around the Bay.

Assessing habitat suitability of a release site is key, as it is perhaps the most important factor determining the success of a reintroduction (McCarthy, Armstrong & Runge, 2012). As an example of the importance of suitable habitat, initial attempts to reestablish the hihi (*Notiomystis cincta*), an endangered New Zealand forest bird, failed due to lack of adequate food resources in the release habitat (Armstrong et al., 1999). Subsequent reintroductions using adaptive management were able to manipulate habitat suitability through supplemental feeding and in doing so achieved success (Armstrong, Castro & Griffiths, 2007). We used a broad definition of suitable habitat based on presence of basic estuarine habitat types, and this resulted in a large study area to work with. With continued research into fine-scale estuarine habitat preferences of sea otters (particularly of marsh habitat-use; *i.e.,* tidal channel order, bank slope, channel width (Espinosa, 2018)) we can refine our assessment of sea otter habitat presence in San Francisco Bay to more accurately assess site suitability. In addition, incorporating finescale abundance and distribution of vital prey resources are going to be crucial. An important area of further research will be to document the spatial distribution and abundance of sea otter prey in San Francisco Bay, and determine whether this coincides with our predicted areas of elevated risk. Decades of study on southern sea otter movement ecology from tagging programs have found that sea otter movement varies by sex and reproductive status, as well as by resource availability. Where resources are limited and otter population more dense, sea otters tend to occupy larger individual homeranges as they likely need to search over a larger area and for a longer amount of time to find sufficient food (Tarjan & Tinker, 2016). While sea otters may generally avoid high risk or highly trafficked parts of the Bay for most of their behaviors, where prey resources abundance overlaps with high risk areas it will necessitate that sea otters are exposed to those risks while foraging.

The ultimate success or failure of any reintroduction will depend on the suitability of the release site to meet the needs of the species. Guidelines for species reintroductions set by the International Union for the Conservation of Nature (IUCN) Species Survival Commission advocate that a reintroduction site must meet the needs of a species not just in the present but through anticipated environmental and climate changes in the future as well (IUCN/SSC, 2013). Therefore, the long-term capacity of San Francisco Bay to provide habitat for sea otters, particularly the effects of sea level rise threatening the viability of saltmarsh, must be taken into consideration. Low-lying portions of the San Francisco Bay shoreline are already experiencing localized flooding during winter storms and extreme tide events (http://www.adaptingtorisingtides.org). These perennial conditions are predicted to become more regular and impactful in the coming decades (Shirzaei & Burgmann, 2018). Conversely, ongoing and planned future tidal marsh restoration throughout the San Francisco Bay is estimated to add over 100,000 acres of saltmarsh to the San Francisco Bay (https://www.sfbayjv.org/). These marsh restoration projects present exciting avenues for the enhancement and expansion of sea otter habitat within the Bay. Our analysis operated under present-day conditions, but it will be important to factor changes to habitat suitability and availability in the future, both positive and negative.

In addition to the changes in physical habitat, managers will need to consider the changes in the presences of the anthropogenic stressors, as these are likely to change over time. For example, the recent completion of a ferry terminal in Richmond, California has added to the volume of ferries on the Bay and created new routes where ferries transit (https://weta.sanfranciscobayferry.com/richmond-ferry-terminal-project). Some anthropogenic stressors might decrease in spatial extent or duration in the future, however. For the first time in a decade the commercial herring fishery did not operate during the 2019 season, due to an unsustainable decline in market value for the fish (https://www.sfchronicle.com/food/article/Commercial-herring-catch-in-SF-Bay-canceled-this-13545808.php). The constantly evolving nature of human use of the Bay makes predicting the resulting risk landscape difficult but nonetheless important for planning species reintroduction.

Much work is still needed in determining suitability of San Francisco Bay for sea otter reintroduction beyond the role of direct threats from anthropogenic activities, such as the availability of sufficient prey resources for sea otters and the potential economic costs and benefits of sea otter recolonization in San Francisco Bay. IUCN guidelines also stress that species reintroductions are not always complementary with local social, political, and economic interests. We encourage decision-makers to consider these diverse interests and engage with stakeholders about the potential benefits and costs of sea otter reintroduction and to identify solutions where possible that maximize benefits and minimize potential tradeoffs that could result in conflicts.

## Conclusions

The spatial patterns of human activities and associated risk in San Francisco Bay that we revealed through our analysis provide answers to important questions and highlight further lines of inquiry about the ability of a highly urbanized estuary to support the recovery of a threatened marine mammal. Spatially explicit maps of risk throughout San Francisco Bay indicated anthropogenic threats are not distributed uniformly. The role that anthropogenic activities play in disrupting connectivity between resource areas and impairing movement of animals both within San Francisco Bay and between San Francisco Bay and outer coast areas requires further consideration. Our approach has demonstrated the use of the HRA as a useful tool for assessing anthropogenic risk across a large geographic area and provides decision-makers with key information to help meet their conservation goals. The HRA allowed us to identify low-risk areas of San Francisco Bay where managers can focus reintroduction efforts as well as areas of concern where anthropogenic threats would be greatest and require additional management actions or contingency plans to mitigate potential harmful interactions. Spatial risk analysis offers a method of rapidly evaluating an urban ecosystem’s strengths and challenges, which can provide valuable information to the planning stages of reintroductions and other types of conservation translocations.

##  Supplemental Information

10.7717/peerj.10241/supp-1 Supplemental Information 1 Spatial footprints of anthropogenic stressors Spatial extent of the seven anthropogenic stressors, defined using data of vessel traffic, contaminants, and fisheries available open-source as listed in Table 1. This was inputted into the Habitat Risk Assessment as the “stressors” component.

10.7717/peerj.10241/supp-2 Supplemental Information 2 Spatial footprint of study area Spatial extent of the study area, defined using presence of suitable sea otter habitat types and water depth. This was used by the Habitat Risk Assessment as the “habitat” component onto which stressors were overlaid.

10.7717/peerj.10241/supp-3 Supplemental Information 3 Key Informant Interview Responses Questions and responses from expert opinion surveys used to determine the weights for the stressors. These were used primarily to inform the intensity criteria, and provided helpful information for determining management effectiveness criteria scores as well.

10.7717/peerj.10241/supp-4 Supplemental Information 4 AIS Vessel Types and Codes A key provided by U.S. Coast Guard, NOAA, BOEM for describing vessel classes and codes found in data collected by Automatic Identification Systems (AIS). This table provides the names of different vessel types, their corresponding Vessel Type and Vessel AIS code, and their classification. In our study, vessel traffic data was extracted using these codes for the vessel types we were interested in (cargo, tanker, passenger, sailing/pleasurecraft.) Some of these were later grouped into similar categories (e.g. cargo and tanker ships become “commercial shipping”).

10.7717/peerj.10241/supp-5 Supplemental Information 5 Study area and stressors ratings The ratings CSV generated by the Habitat Risk Assessment preprocessor tool, with final weighting scores for each stressor in each of the categories that the HRA uses to calculate risk applied. Scores were determined using expert opinion surveys.

10.7717/peerj.10241/supp-6 Supplemental Information 6 Code for generating exposure-consequence risk plots

## Additional Information and Declarations

### Data Availability

The following information was supplied regarding data availability: Data, anonymous expert opinion interview notes, and code are available in the Supplemental Files.
