# Supplementary material for: Assessing anthropogenic risk to sea otters (Enhydra lutris nereis) for reintroduction into San Francisco Bay
Source: PeerJ. 2020 Nov 17;8:e10241. doi: 10.7717/peerj.10241 (PMC7678461; doi:10.7717/peerj.10241)
Supplement: Supplemental Information 4 — A key provided by U.S. Coast Guard, NOAA, BOEM for describing vessel classes and codes found in data collected by Automatic Identification Systems (AIS). This table provides the names of different vessel types, their corresponding Vessel Type and Vessel AIS code, and their classification. In our study, vessel traffic data was extracted using these codes for the vessel types we were interested in (cargo, tanker, passenger, sailing/pleasurecraft.) Some of these were later grouped into similar categories (e.g. cargo and tanker ships become “commercial shipping”). [file peerj-08-10241-s004.pdf]

Vessel Group (2018) key

- Cargo
- Fishing
- Military
- Not Available
- Other
- Passenger
- Pleasure Craft/Sailing
- Tanker
- Tug Tow

AIS Vessel Type and Group Codes used by the Marine Cadastre Project
2018 -05-23

sources: U.S. Coast Guard, NOAA, BOEM

Vessel Group (2018)	Vessel Type (2018)	AIS Vessel Code	AIS Ship & Cargo Classification
Not Available	0	0	Not available or no ship, default
Other	1-19	1-19	Reserved for future use
Other	20	20	Wing in ground (WIG), all ships of this type
Tug Tow	21	21	Wing in ground (WIG), hazardous category A
Tug Tow	22	22	Wing in ground (WIG), hazardous category B
Other	23	23	Wing in ground (WIG), hazardous category C
Other	24	24	Wing in ground (WIG), hazardous category D
Other	25	25	Wing in ground (WIG), reserved for future use
Other	26	26	Wing in ground (WIG), reserved for future use
Other	27	27	Wing in ground (WIG), reserved for future use
Other	28	28	Wing in ground (WIG), reserved for future use
Other	29	29	Wing in ground (WIG), reserved for future use
Fishing	30	30	Fishing
Tug Tow	31	31	Towing
Tug Tow	32	32	Towing: length exceeds 200m or breadth exceeds 25m
Other	33	33	Dredging or underwater operations
Other	34	34	Diving operations
Military	35	35	Military operations
Pleasure Craft/Sailing	36	36	Sailing
Pleasure Craft/Sailing	37	37	Pleasure Craft
Other	38	38	Reserved
Other	39	39	Reserved
Other	40	40	High speed craft (HSC), all ships of this type
Other	41	41	High speed craft (HSC), hazardous category A
Other	42	42	High speed craft (HSC), hazardous category B
Other	43	43	High speed craft (HSC), hazardous category C
Other	44	44	High speed craft (HSC), hazardous category D
Other	45	45	High speed craft (HSC), reserved for future use
Other	46	46	High speed craft (HSC), reserved for future use
Other	47	47	High speed craft (HSC), reserved for future use
Other	48	48	High speed craft (HSC), reserved for future use
Other	49	49	High speed craft (HSC), no additional information
Other	50	50	Pilot Vessel
Other	51	51	Search and Rescue vessel
Tug Tow	52	52	Tug
Other	53	53	Port Tender
Other	54	54	Anti-pollution equipment
Other	55	55	Law Enforcement
Other	56	56	Spare - for assignment to local vessel
Other	57	57	Spare - for assignment to local vessel
Other	58	58	Medical Transport
Other	59	59	Ship according to RR Resolution No. 18

	Passenger	60	60	Passenger, all ships of this type
	Passenger	61	61	Passenger, hazardous category A
	Passenger	62	62	Passenger, hazardous category B
	Passenger	63	63	Passenger, hazardous category C
	Passenger	64	64	Passenger, hazardous category D
	Passenger	65	65	Passenger, reserved for future use
	Passenger	66	66	Passenger, reserved for future use
	Passenger	67	67	Passenger, reserved for future use
	Passenger	68	68	Passenger, reserved for future use
	Passenger	69	69	Passenger, no additional information
	Cargo	70	70	Cargo, all ships of this type
	Cargo	71	71	Cargo, hazardous category A
	Cargo	72	72	Cargo, hazardous category B
	Cargo	73	73	Cargo, hazardous category C
	Cargo	74	74	Cargo, hazardous category D
	Cargo	75	75	Cargo, reserved for future use
	Cargo	76	76	Cargo, reserved for future use
	Cargo	77	77	Cargo, reserved for future use
	Cargo	78	78	Cargo, reserved for future use
	Cargo	79	79	Cargo, no additional information
	Tanker	80	80	Tanker, all ships of this type
	Tanker	81	81	Tanker, hazardous category A
	Tanker	82	82	Tanker, hazardous category B
	Tanker	83	83	Tanker, hazardous category C
	Tanker	84	84	Tanker, hazardous category D
	Tanker	85	85	Tanker, reserved for future use
	Tanker	86	86	Tanker, reserved for future use
	Tanker	87	87	Tanker, reserved for future use
	Tanker	88	88	Tanker, reserved for future use
	Tanker	89	89	Tanker, no additional information
	Other	90	90	Other Type, all ships of this type
	Other	91	91	Other Type, hazardous category A
	Other	92	92	Other Type, hazardous category B
	Other	93	93	Other Type, hazardous category C
	Other	94	94	Other Type, hazardous category D
	Other	95	95	Other Type, reserved for future use
	Other	96	96	Other Type, reserved for future use
	Other	97	97	Other Type, reserved for future use
	Other	98	98	Other Type, reserved for future use
	Other	99	99	Other Type, no additional information
	Other	100 to 199	100 to 199	Reserved for regional use
	Other	200 to 255	200 to 255	Reserved for future use
	Other	256 to 999	256 to 999	No designation

	Vessel Group (2018)	VesselType (2018)		AVIS Vessel Service
	Other	-	-	null
	Fishing	1001	-	Commercial Fishing Vessel
	Fishing	1002	-	Fish Processing Vessel
	Cargo	1003	-	Freight Barge
	Cargo	1004	-	Freight Ship
	Other	1005	-	Industrial Vessel
	Other	1006	-	Miscellaneous Vessel
	Other	1007	-	Mobile Offshore Drilling Unit
	Other	1008	-	Non-vessel

	Other	1009	-	NON-VESSEL
	Other	1010	-	Offshore Supply Vessel
	Other	1011	-	Oil Recovery
	Passenger	1012	-	Passenger (Inspected)
	Passenger	1013	-	Passenger (Uninspected)
	Passenger	1014	-	Passenger Barge (Inspected)
	Passenger	1015	-	Passenger Barge (Uninspected)
	Cargo	1016	-	Public Freight
	Tanker	1017	-	Public Tankship/Barge
	Other	1018	-	Public Vessel, Unclassified
	Pleasure Craft/Sailing	1019	-	Recreational
	Other	1020	-	Research Vessel
	Military	1021	-	SAR Aircraft
	Other	1022	-	School Ship
	Tug Tow	1023	-	Tank Barge
	Tanker	1024	-	Tank Ship
	Tug Tow	1025	-	Towing Vessel
